# Trastuzumab deruxtecan for the treatment of metastatic non-small cell lung cancer harboring *HER2* non-exon 19/20 mutations: four case reports

**DOI:** 10.3389/fimmu.2025.1631768

**Published:** 2025-08-12

**Authors:** Yan Meng, Yanping Du, Xiaolin Liu, Jue Huang, Hanhan Chen, Chunxia He

**Affiliations:** ^1^ Department of Medical Oncology, 2nd District, Hainan Cancer Hospital, Hainan, China; ^2^ The School of Clinical Medicine, Fujian Medical University, Fuzhou,, China; ^3^ Department of Pulmonary and Critical Care Medicine, Zhongshan Hospital Xiamen University, School of Medicine, Xiamen University, Xiamen, China; ^4^ Guangzhou University of Chinese Medicine, Guangzhou, China; ^5^ Guangdong Provincial Hospital of Chinese Medicine, The Second Clinical Medical College, University of Guangzhou Traditional Chinese Medicine, Guangzhou, China

**Keywords:** trastuzumab deruxtecan, non-small cell lung cancer, human epidermal growth factor receptor 2, mutation, case series

## Abstract

**Background:**

Patients with *human epidermal growth factor receptor 2* (*HER2*)-mutant non-small cell lung cancer (NSCLC) have poor prognosis. Trastuzumab deruxtecan (T-DXd) is the first targeted therapy approved for the treatment of patients with *HER2*-mutant metastatic NSCLC, but the evidence in those with *HER2* non-exon 19/20 mutations is scarce.

**Methods:**

We reported treatment information and outcomes of four patients with metastatic NSCLC harboring *HER2* non-exon 19/20 mutations who were treated with T-DXd.

**Results:**

All the four patients had metastatic lung adenocarcinoma and reached partial response to T-DXd treatment. A 57-year-old female patient with *HER2* exon 17 V659E mutation received T-DXd as later-line treatment. Treatment was ongoing and the progression-free survival (PFS) had reached 13 months. Three patients received first-line T-DXd treatment. One patient with *HER2* exon 3 T126A mutation had disease progression after 16-month treatment. The other two patients (one with *HER2* exon 21 H878Y mutation and one with *HER2* exon 17 V659E mutation) were continuing T-DXd treatment, both with a PFS of more than 6 months. No interstitial lung disease or grade ≥3 adverse events occurred in these four patients.

**Conclusion:**

The potential of T-DXd in patients with metastatic NSCLC harboring *HER2* non-exon 19/20 mutations is considerable, which deserves to be validated in large-sample studies.

## Introduction

Lung cancer is the most prevalent cancer and the leading cause of cancer death worldwide, with 2.48 million new cases and 1.82 million deaths in 2022 (1). The majority of lung cancer patients (85%) had non-small cell lung cancer (NSCLC) (2). *Human epidermal growth factor receptor 2* (*HER2*) mutations exist in approximately 2% of NSCLC cases (3), which are associated with poor prognosis. The predominant locations of *HER2* mutations are exon 20 and exon 19, while *HER2* non-exon 19/20 mutations only account for approximately 20% of *HER2*-mutant NSCLC cases (4). The preferred first-line treatment option for *HER2*-mutant metastatic NSCLC refers to that for metastatic NSCLC without driver oncogenes (platinum-based chemotherapy with or without immunotherapy) (5, 6). After progression, single-agent chemotherapy or immunotherapy can be selected. More and more biomarker researches are conducted to predict treatment efficacy and assist decision-making, especially in the era of immunotherapy (7–9). Nevertheless, these therapies provide limited benefits for patients with *HER2*-mutant advanced NSCLC (10, 11), reflecting the unmet need for effective therapies in this population.

Despite the great development of anti-HER2 drugs in the past decades, their effects in *HER2*-mutant NSCLC are not optimistic. Until August 2022, the anti-HER2 antibody-drug conjugate trastuzumab deruxtecan (T-DXd) became the first targeted therapy approved by the United States Food and Drug Administration for the treatment of patients with metastatic NSCLC harboring *HER2* mutations based on the unprecedented efficacy and manageable safety profile (12). The final analysis of DESTINY-Lung02 showed an objective response rate of 50.0%, a median progression-free survival (PFS) of 10.0 months, and a median overall survival of 19.0 months with later-line T-DXd treatment at the approved dose (5.4 mg/kg once every 3 weeks) (13). Recently, this indication was also approved by China National Medical Products Administration in October 2024. The 2025 Chinese Society of Clinical Oncology guideline has endorsed T-DXd as later-line therapy (Grade I recommendation). However, previous studies mainly focused on patients with *HER2* exon 19/20 mutations, while the evidence in those with *HER2* non-exon 19/20 mutations is scarce.

Here we reported the treatment information and outcomes of four patients with metastatic NSCLC harboring *HER2* non-exon 19/20 mutations who were treated with T-DXd (**Table 1**).

**Table 1:** Characteristics of patients reported in this case series.

Reference	Age (years)	Sex	Smoking history	Histology	Stage	*HER2* alterations	Prior treatment lines	Treatment	Best response	PFS (months)
Case 1	57	F	No	Ad	IV	Exon 17 V659E; amplification	3	T-DXd, 18+ cycles	PR	13+
Case 2	59	M	No	Ad	IV	Exon 21 H878Y	0	T-DXd, 9+ cycles	PR	7+
Case 3	75	F	No	Ad	IV	Exon 17 V659E	0	T-DXd, 10+ cycles	PR	8+
Case 4	81	M	Yes	Ad	IV	Exon 3 T126A; amplification	0	T-DXd, 18 cycles	PR	16

T-DXd, trastuzumab deruxtecan; *HER2*, *human epidermal growth factor receptor 2*; PFS, progression-free survival; F, female; Ad, adenocarcinoma; PR, partial response; M, male; PD, progressive disease; MR, metabolic response.

## Case series

### Case 1

A 57-year-old female was admitted in May 2020 due to severe pain in the shoulder. The patient was diagnosed with lung adenocarcinoma in the right lower lobe with mediastinal lymph nodes, liver, and bone metastases in July 2019. She refused treatment at that time. Gene detection showed no *epidermal growth factor receptor* (*EGFR*), *anaplastic lymphoma kinase* (*ALK*), or *ROS proto-oncogene 1* (*ROS1*) alterations, and the programmed cell death-ligand 1 (PD-L1) tumor proportion score (TPS) was 3%.

**Figure 1:**
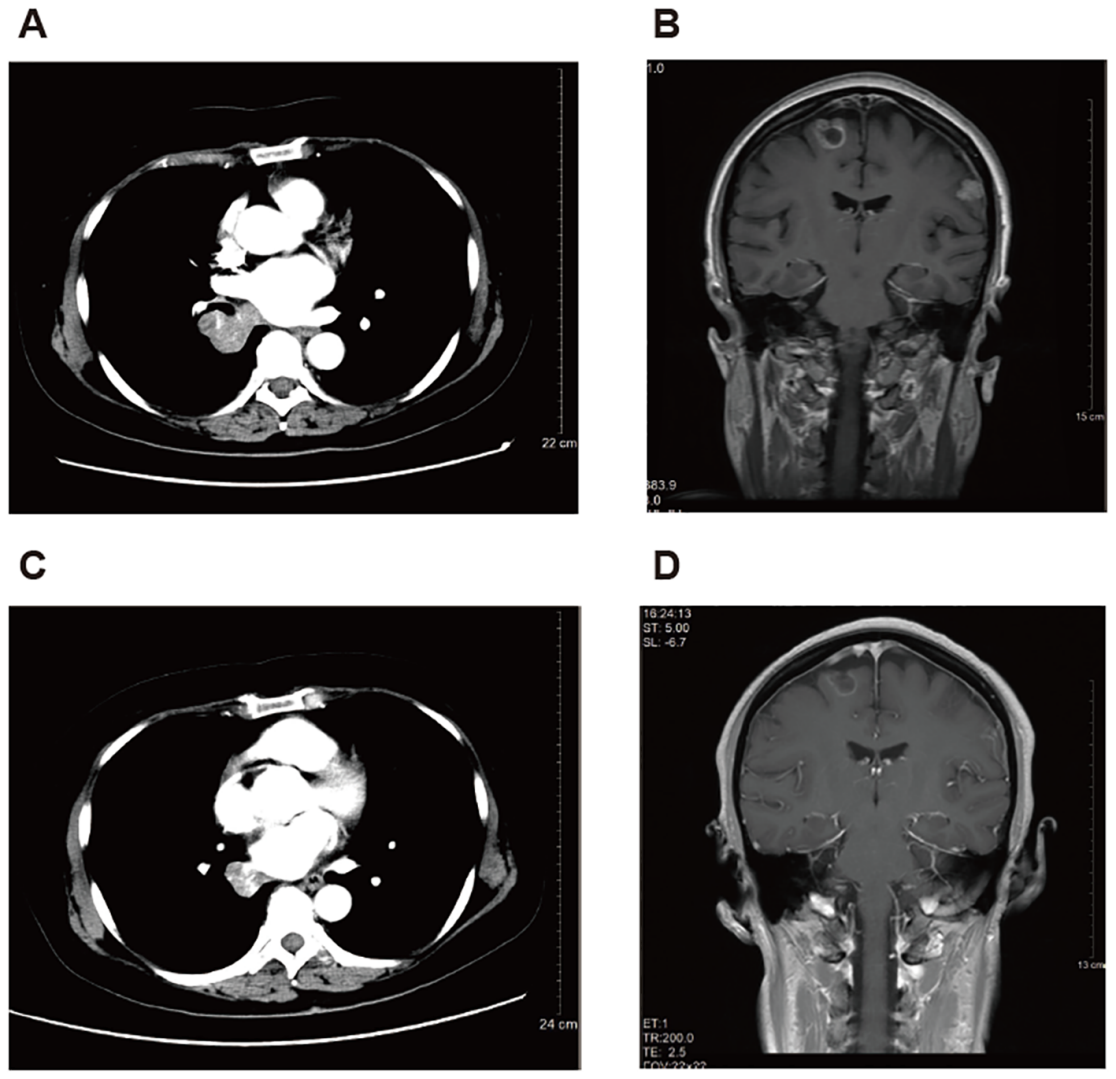
Representative images of computed tomography in a 57-year-old female patient with metastatic non-small cell lung cancer harboring *HER2* exon 17 V659E mutation and amplification treated with later-line trastuzumab deruxtecan. **(A, B)** Before treatment of trastuzumab deruxtecan. **(C, D)** After 10-month treatment of trastuzumab deruxtecan.

At this admission, the imaging examinations indicated that the tumor progressed to the middle lobe of the right lung and spread to the brain. The disease stage was cT4N2M1c. The patient received tislelizumab (200 mg, day 1) plus pemetrexed (750 mg, day 1), carboplatin (400 mg, day 1), and bevacizumab (600 mg, day 1) for six 21-day cycles since May 2020, but refused local radiotherapy for brain and bone lesions. In November 2020, computed tomography (CT) and magnetic resonance imaging (MRI) indicated partial response (PR). Then she received maintenance therapy with tislelizumab (200 mg, day 1) plus bevacizumab (400 mg, day 1). In May 2021, CT indicated progression of lung lesions, with stable liver and brain lesions. She continued another 4 cycles of tislelizumab plus bevacizumab with the addition of albumin-bound paclitaxel (400 mg, day 1) and carboplatin (400 mg, day 1). In September 2021, CT indicated that the lung lesions reached PR, with stable liver and brain lesions. Then she received maintenance therapy with tislelizumab plus bevacizumab and vinorelbine (40 mg, days 1, 3, and 5 every week) until March 2023, and subsequently received bevacizumab (300 mg, day 1) plus vinorelbine (40 mg, days 1, 3, and 5 every week). In June 2023, CT indicated disease progression. Vinorelbine was switched to gemcitabine (1400 mg, days 1 and 8) plus cisplatin (90 mg, day 1) while bevacizumab treatment was continued. In August 2023, CT and MRI indicated stable lung and brain lesions (**Figures 1A, B**). The biopsy showed that the patient had microsatellite-stable, PD-L1-negative disease with *HER2* mutation (exon 17 V659E) and *HER2* amplification. Then she began to receive T-DXd (5.4 mg/kg once every 3 weeks) monotherapy. She had a PR in June 2024 (**Figures 1C, D**), which was sustained by the last follow-up date (September 2024). Nausea, vomiting, increased creatinine, decreased white blood cell count, and elevated liver enzymes occurred during T-DXd treatment, which were all grade 1-2 and could be managed with symptomatic treatments.

### Case 2

A 59-year-old male was admitted in February 2024 due to incidental findings of elevated tumor markers (carcinoembryonic antigen [CEA]: 57.57 ng/mL; CA-199: 323.14 U/mL) from a health examination. CT indicated lung adenocarcinoma in the right upper lobe (21×16 mm) with mediastinal, retroperitoneal and inguinal lymph nodes (maximum short diameter: 14 mm), and adrenal gland (21×17 mm) metastases. Emission CT (ECT) indicated bone metastases. The disease stage was cT1cN2bM1c2. Gene detection showed *HER2* (exon 21 H878Y) and *TP53* (S241C) mutations, and the PD-L1 TPS was 20%. Physical examinations, previous medical history, and laboratory tests were unremarkable. The patient received T-DXd (5.0 mg/kg once every 3 weeks) as first-line treatment since March 2024. In April 2024, CT indicated that he had a PR, which was sustained by the last follow-up date (October 2024). No treatment-related adverse events occurred during T-DXd treatment.

### Case 3

A 75-year-old female was admitted in November 2023 due to cough, expectoration, and asthma for at least one month. Enhanced CT indicated massive left pleural effusion, left lung atelectasis, inflammation in the upper lobe of the left lung, space-occupying lesions in the lower lobe of the left lung, left hilar lymph nodes enlargement, multiple nodules in bilateral lungs, solid nodules in the lower lobe of the right lung, and thickened left pleura. Lung adenocarcinoma was confirmed by the cytological examination of pleural effusion, and the disease stage was TxNxM1. The next-generation sequencing of pleural effusion showed *HER2* mutation (exon 17 V659E). Physical examinations, previous medical history, and laboratory tests were unremarkable. The patient received T-DXd (5.4 mg/kg once every 3 weeks) as first-line treatment since December 2023. Clinical symptoms such as asthma were gradually improved after treatment. In March 2024, CT indicated that she had a PR, which was sustained by the last follow-up date (August 2024). Nausea, vomiting, anemia, increased aspartate aminotransferase, and increased direct bilirubin occurred during T-DXd treatment, which were all grade 1-2 and could be managed with symptomatic treatments.

### Case 4

An 81-year-old male was admitted in March 2023 for the treatment of metastatic NSCLC. The patient was diagnosed with poorly-differentiated lung adenocarcinoma in the left lower lobe in June 2020 and underwent thoracoscopic-assisted radical resection. The pathological stage was T2aN1M0. After surgery, he received adjuvant chemotherapy with pemetrexed (700 mg, day 1) plus nedaplatin (100 mg, day 2) for two 21-day cycles from July 2020 to August 2020, and discontinued due to intolerable toxicity. In February 2023, CT indicated metastatic tumor lesions in bilateral lungs. He had coronary heart disease and severe hypertension, and had smoking history for 70 years.

**Figure 2:**
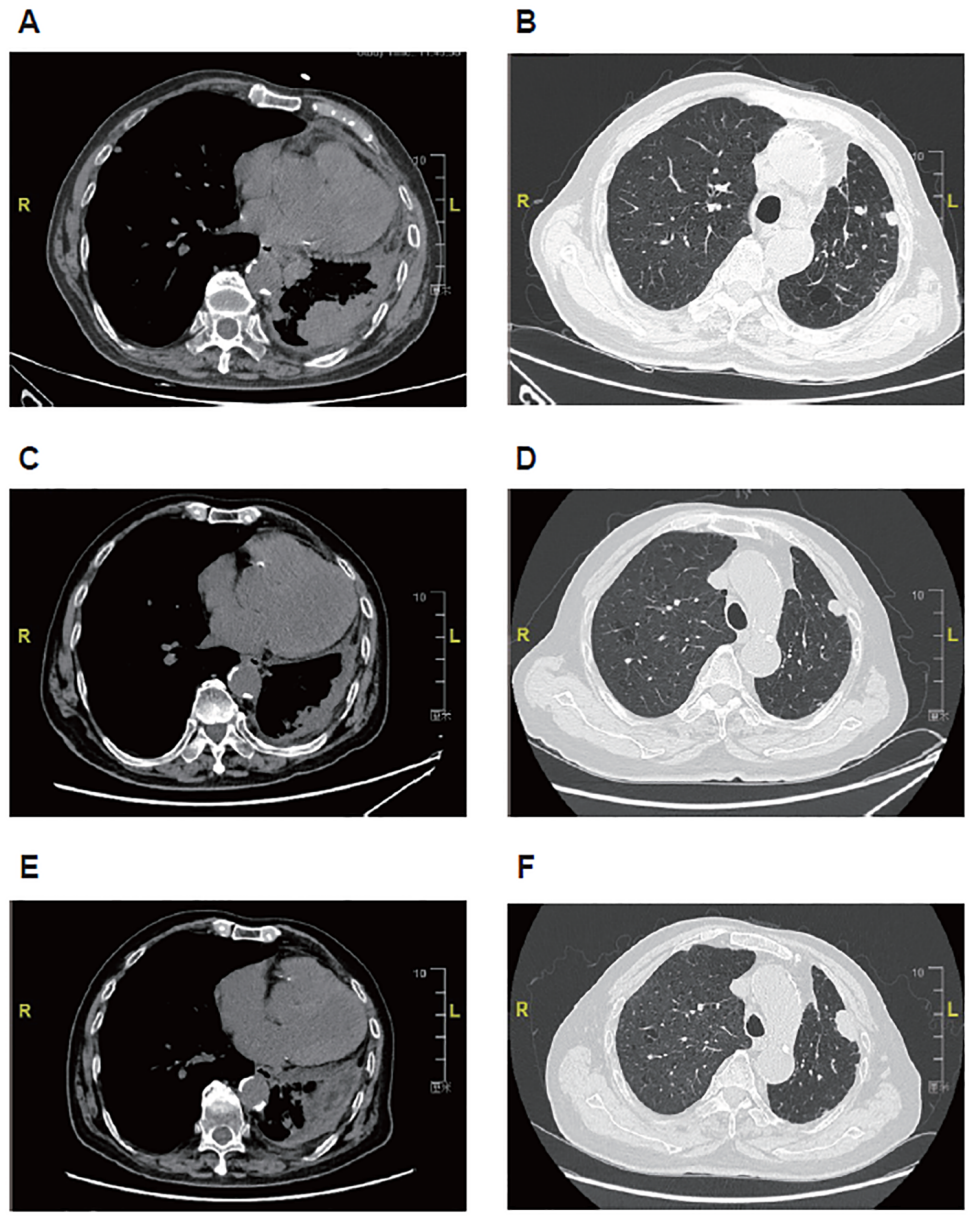
Representative images of computed tomography in an 81-year-old male patient with metastatic non-small cell lung cancer harboring *HER2* exon 3 T126A mutation and amplification treated with first-line trastuzumab deruxtecan. **(A, B)** Before treatment of trastuzumab deruxtecan. **(C, D)** After 12-month treatment of trastuzumab deruxtecan. **(E, F)** After 16-month treatment of trastuzumab deruxtecan.

At this admission, the physical examinations and laboratory tests showed complete right bundle branch block, hemoglobin 78 g/L, Eastern Cooperative Oncology Group (ECOG) performance status of 3, and no other remarkable findings. CT indicated multiple nodules (the largest one with obscure boundary: 49×28 mm) in bilateral lungs, inflammation accompanied with lymphangitis carcinomatosa in the residual left lung, multiple lymph node metastases (supraclavicular fossa, crural diaphragm, left heart diaphragmatic angle, right hilum, and mediastinum; maximum short diameter: 23 mm), and thickened left pleura (**Figures 2A, B**). The disease stage was rpT4N3M1a. Gene detection using the surgically resected sample showed *HER2* mutation (exon 3 T126A) and *HER2* amplification. The patient received T-DXd (3.4 mg/kg once every 3 weeks) since March 2023, with the best response of PR (**Figures 2C, D**) and improvement in ECOG performance status (from 3 to 1). Nausea and asthenia occurred during 18 cycles of T-DXd monotherapy, which were both grade 2. In July 2024, color ultrasound indicated left supraclavicular lymph node metastases and CT indicated disease progression (**Figures 2E, F**). Then anlotinib (10 mg once daily) was added to T-DXd. After one 21-day cycle, T-DXd was switched to pemetrexed (700 mg, day 1) due to poor tolerability, while anlotinib treatment was continued.

## Discussion

Variation in *HER2* mutation locations is likely to affect sensitivity to anti-HER2 therapy (14). Owing to the rarity of *HER2* non-exon 19/20 mutations, reliable evidence and well-established treatment options are still lacking. Despite that T-DXd has been approved for treating *HER2*-mutant metastatic NSCLC, its efficacy in patients with *HER2* non-exon 19/20 mutations is not fully revealed. The pivotal DESTINY-Lung01 and DESTINY-Lung02 trials mainly included patients with *HER2* exon 19/20 mutations (15, 16). Previous case reports of T-DXd published individual data in *HER2* exon 20 mutant NSCLC only (17–24). We supplemented specific efficacy and safety data in this clinical scenario, which can provide some reference for clinicians.

Patient 1 had advanced lung adenocarcinoma at diagnosis, accompanied with multiple organ metastases. The patient had no driver oncogenes, and the PD-L1 TPS was 3%. Considering that the patient was relatively young, the four-drug combination of immunotherapy plus chemotherapy and antiangiogenic therapy was administered. Despite that the chemotherapeutic agents were switched after each disease progression, the patient experienced treatment failure twice in 3 years. During the third-line treatment with chemotherapy plus antiangiogenic therapy, *HER2* mutation was detected by biopsy. Although the patient had rare exon 17 V659E mutation rather than exon 19/20 mutations, she still received T-DXd monotherapy, with the best response of PR. As a later-line treatment, the PFS of at least 13 months is definitely encouraging. A total of eight evaluable patients with previously treated metastatic NSCLC and *HER2* non-exon 19/20 mutations were enrolled in DESTINY-Lung01 and DESTINY-Lung02 (15, 16). Three (38%) of them achieved a PR to T-DXd treatment and four achieved stable disease with shrunken lesions (15, 16), indicating good disease control. Regarding other anti-HER2 therapies, some previous studies explored pan-HER tyrosine kinase inhibitors in this population. The phase 2 trial by Song et al. investigated pyrotinib in patients with advanced lung adenocarcinoma and *HER2* mutations, and 70.5% of patients received pyrotinib as second- or further-line treatment (25). A total of ten patients with *HER2* non-exon 19/20 mutations were included in this trial, but only one patient with exon 17 mutation had a PR to pyrotinib treatment, with an objective response rate of 10% (25). In a study by Ou et al., one patient with metastatic lung adenocarcinoma and *HER2* exon 17 mutations (V659E/G660R) achieved a PR to second-line afatinib treatment with a PFS of at least 18 months (26). Another patient with metastatic lung adenocarcinoma and peritoneal metastases harboring *HER2* exon 17 V659E mutation achieved significant symptom improvement and a metabolic response after 3-month afatinib treatment (26). However, one patient with metastatic lung adenocarcinoma and *HER2* exon 17 G660D mutation had a rapid disease progression after 10 weeks of second-line afatinib treatment (26). All these results suggest the potential of T-DXd for the treatment of patients with metastatic NSCLC and *HER2* non-exon 19/20 mutations in the later-line setting, which warrants further validation. Given the small sample size, whether the clinical benefits following T-DXd treatment in patients with *HER2* non-exon 19/20 mutations are consistent or different compared with the *HER2* exon 19/20 mutant population also needs further investigation.

The other three patients in our case series received T-DXd treatment in the first-line setting, and all achieved a PR. Notably, patient 4 achieved a PFS of 16 months before progression on T-DXd treatment. Although the follow-up duration was too short to reflect the long-term survival benefits for patients 2 and 3, they had a rapid response after 1-3 months of T-DXd treatment. The international, randomized controlled, phase 3 DESTINY-Lung04 trial (NCT05048797) is ongoing to prove the role of T-DXd as first-line treatment in patients with advanced NSCLC and *HER2* exon 19/20 mutations. Our results preliminarily showed the promising value of first-line T-DXd treatment in those with *HER2* non-exon 19/20 mutations, which deserves to be further investigated. In the study by Ou et al., one patient with metastatic lung adenocarcinoma and bone metastases harboring *HER2* exon 17 V659E mutation rapidly responded to afatinib after 1 month of first-line treatment, but the follow-up time was inadequate (26). No more first-line data were disclosed for afatinib or other anti-HER2 therapies in patients with metastatic NSCLC and *HER2* non-exon 19/20 mutations.

The adverse events profiles in these four patients were acceptable. No interstitial lung disease or grade ≥3 adverse events occurred. With the wide application of T-DXd, the monitoring and prevention of adverse events will become more standardized. There will be less concern about its toxicity risk in clinical practice. For two elderly patients aged ≥75 years, only mild gastrointestinal reactions, anemia, asthenia, and hepatobiliary toxicities were observed. The 81-year-old patient even had largely improved ECOG performance status after T-DXd treatment, indicating its feasibility in elderly patients who are probably intolerable to chemotherapy.

There are some limitations in this case series. First, the sample size was indeed too small to draw any convincing conclusions. Second, the follow-up duration was short in two of four patients. Large-scale prospective evidence with long-term follow-up is urgently needed to validate our findings.

In conclusion, our case series preliminarily shows the promising efficacy and acceptable safety profile of T-DXd in patients with metastatic NSCLC harboring *HER2* non-exon 19/20 mutations. These findings warrant further large-sample validation.

## Data Availability

The original contributions presented in the study are included in the article/supplementary material. Further inquiries can be directed to the corresponding authors.
