# Supplementary material for: Trastuzumab deruxtecan for the treatment of metastatic non-small cell lung cancer harboring HER2 non-exon 19/20 mutations: four case reports
Source: Front Immunol. 2025 Aug 12;16:1631768. doi: 10.3389/fimmu.2025.1631768 (PMC12378165; doi:10.3389/fimmu.2025.1631768)
Supplement: Supplementary file 1 [file DataSheet1.pdf]

CARE case report guidelines

CARE Checklist of information to include when writing a case report

(cc) BY-NC-ND

Topic	Item	Checklist item description	Reported on Line
Title	1	The diagnosis or intervention of primary focus followed by the words "case report"	1-2
Key Words	2	2 to 5 key words that identify diagnoses or interventions in this case report, including "case report"	45-46
Abstract (no references)	3a	Introduction: What is unique about this case and what does it add to the scientific literature?	30-33
	3b	Main symptoms and/or important clinical findings	33-35
	3c	The main diagnoses, therapeutic interventions, and outcomes	35-42
	3d	Conclusion—What is the main "take-away" lesson(s) from this case?	42-44
Introduction	4	One or two paragraphs summarizing why this case is unique (**may include references**)	48-76
Patient Information	5a	De-identified patient specific information.	80-81, 113-114, 126-127, 143
	5b	Primary concerns and symptoms of the patient.	86-87, 115-120, 144-147, 154-160
	5c	Medical, family, and psycho-social history including relevant genetic information	81-83, 120-121, 150-151
	5d	Relevant past interventions with outcomes	81-85, 146-149
Clinical Findings	6	Describe significant physical examination (PE) and important clinical findings.	86, 127-131, 152-154
Timeline	7	Historical and current information from this episode of care organized as a timeline	86-111, 115-123, 135-138
Diagnostic Assessment	8a	Diagnostic testing (such as PE, laboratory testing, imaging, surveys).	86-88, 121-123, 154-159
	8b	Diagnostic challenges (such as access to testing, financial, or cultural)	91-92
	8c	Diagnosis (including other diagnoses considered)	83-87, 152-160
	8d	Prognosis (such as staging in oncology) where applicable	87-88, 121-134, 159-160
Therapeutic Intervention	9a	Types of therapeutic intervention (such as pharmacologic, surgical, preventive, self-care)	88-91, 121-122, 135-136, 144-146, 161-164
	9b	Administration of therapeutic intervention (such as dosage, strength, duration)	88-103, 121-122, 135-136
	9c	Changes in therapeutic intervention (with rationale)	106-107, 166-169
Follow-up and Outcomes	10a	Clinician and patient-assessed outcomes (if available)	
	10b	Important follow-up diagnostic and other test results	107-108, 122-123, 137-138, 165-166
	10c	Intervention adherence and tolerability (How was this assessed?)	108-111, 167-169
	10d	Adverse and unanticipated events	108-111, 121-124, 138-141
Discussion	11a	A scientific discussion of the strengths AND limitations associated with this case report	225-237
	11b	Discussion of the relevant medical literature **with references**.	210-224
	11c	The scientific rationale for any conclusions (including assessment of possible causes)	179-209
	11d	The primary "take-away" lessons of this case report (without references) in a one paragraph conclusion	238-241
Patient Perspective	12	The patient should share their perspective in one to two paragraphs on the treatment(s) they received	225-237
Informed Consent	13	Did the patient give informed consent? Please provide if requested	Yes ☑ No ☐
